# Discover hidden splicing variations by mapping personal transcriptomes to personal genomes

**DOI:** 10.1093/nar/gkv1099

**Published:** 2015-11-17

**Authors:** Shayna Stein, Zhi-xiang Lu, Emad Bahrami-Samani, Juw Won Park, Yi Xing

**Affiliations:** Department of Microbiology, Immunology, and Molecular Genetics, University of California, Los Angeles, Los Angeles, CA 90095, USA

## Abstract

RNA-seq has become a popular technology for studying genetic variation of pre-mRNA alternative splicing. Commonly used RNA-seq aligners rely on the consensus splice site dinucleotide motifs to map reads across splice junctions. Consequently, genomic variants that create novel splice site dinucleotides may produce splice junction RNA-seq reads that cannot be mapped to the reference genome. We developed and evaluated an approach to identify ‘hidden’ splicing variations in personal transcriptomes, by mapping personal RNA-seq data to personal genomes. Computational analysis and experimental validation indicate that this approach identifies personal specific splice junctions at a low false positive rate. Applying this approach to an RNA-seq data set of 75 individuals, we identified 506 personal specific splice junctions, among which 437 were novel splice junctions not documented in current human transcript annotations. 94 splice junctions had splice site SNPs associated with GWAS signals of human traits and diseases. These involve genes whose splicing variations have been implicated in diseases (such as *OAS1*), as well as novel associations between alternative splicing and diseases (such as *ICA1*). Collectively, our work demonstrates that the personal genome approach to RNA-seq read alignment enables the discovery of a large but previously unknown catalog of splicing variations in human populations.

## INTRODUCTION

Exons can be differentially included in the mature mRNA products during splicing (1). This process, called alternative splicing (AS), is one of the predominant mechanisms for generating distinct mRNA isoforms from a single gene. It is estimated that over 90% of human multi-exon genes are alternatively spliced (2,3). AS plays a crucial role in gene regulation and abnormal variations in splicing can have significant disease consequences (4). In fact, as high as 50% of disease-causing mutations in specific disease genes may alter splicing (5,6).

In recent years, numerous studies have identified single nucleotide polymorphisms (SNPs) that are associated with changes in nearby AS events, commonly referred to as splicing quantitative trait loci (sQTLs) (7–10). Collectively, these studies have established that natural genetic polymorphisms in the human population may cause differences in exon usage patterns or splicing efficiencies among individuals. Such natural variation of alternative splicing may in turn influence disease risk or severity or therapeutic response (11–13). Thus, the discovery of sQTLs will reveal potential mechanisms underlying human phenotypic diversity and susceptibility to genetic disorders.

AS is regulated by a wide array of *cis* regulatory elements on the pre-mRNA as well as *trans*-acting factors that interact with these *cis* elements (14). The most conserved *cis* splicing signals within the pre-mRNA are the 5′ and 3′ splice sites, which define the boundary between exons and introns. Approximately 99% of mammalian splice sites follow the ‘GT-AG’ dinucleotide rule such that the first two and last two nucleotides in the intron are GT and AG, respectively. Of the remaining splice sites, ≈0.9% are ‘GC-AG’ and ≈0.09% are ‘AT-AC’ (15). Genetic variants that disrupt or create the highly conserved splice site dinucleotide motifs can alter splicing patterns and produce alternative mRNA and protein isoforms (16). Indeed, mutations that affect splice site dinucleotides represent a large class of human disease mutations (17).

**Figure 1.**
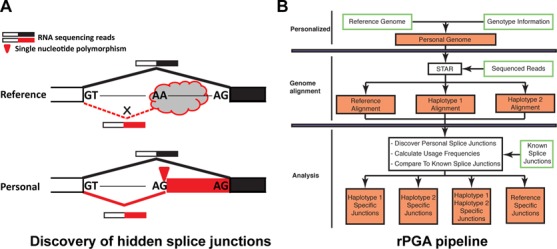
Identifying hidden splice junctions by aligning personal RNA-seq reads to personal genomes. **(A)** RNA-seq splice junction reads originating from SNPs creating personal splice site dinucleotide motifs (shown in red) do not align to the reference genome due to non-canonical splice site motifs in the reference genome. The RNA-seq splice junction reads do, however, align to the personal genome. **(B)** Flowchart of the rPGA pipeline.

RNA sequencing (RNA-seq) has emerged as a powerful method for discovering and quantifying AS events at the whole-transcriptome scale. In a typical RNA-seq data analysis workflow, sequenced fragments of mRNA (i.e. reads) are aligned to the reference genome sequence and/or existing transcript annotations, and reads corresponding to specific exons and splice junctions are identified and counted to generate quantitative estimates of gene expression and alternative splicing (18–21). A number of studies have used this strategy to identify associations between genetic polymorphisms and alternative splicing events in human populations (22–28). However, the use of the reference genome has important limitations for studying individual variations of transcriptomes. For example, it is well known that when mapping reads to the reference genome, exonic SNPs can create a bias for mapping RNA-seq reads harboring the reference alleles over reads harboring the derived alleles, which may skew the quantitation of allelic ratios in RNA-seq data and confound downstream analyses of allele-specific gene expression and RNA processing (29). Methods have been developed to alleviate such biases in mapping personal RNA-seq data (30–32). Another major limitation, which is the main motivation for this work, is the identification of splice junctions from personal RNA-seq reads aligned to the reference genome. Many commonly used RNA-seq aligners, including Tophat and SpliceMap (33,34), rely on the canonical (e.g. GT-AG, GC-AG, AT-AC) splice site dinucleotide motifs and do not align reads to non-canonical splice junctions. Other aligners, such as STAR and HISAT (35,36), apply a severe score penalty to non-canonical splice junction alignments. As a result, if a genetic polymorphism creates a novel splice site dinucleotide motif in an individual, RNA-seq reads that originate from the polymorphic splice site in the personal genome will likely be unmappable to the human reference genome due to the lack of the canonical splice site dinucleotide motif in the reference genome sequence (Figure 1A). This would result in ‘hidden’ splicing variations that are undetected by standard RNA-seq alignment procedures.

In this work, we explored whether a personal genome approach to RNA-seq alignment could detect such hidden splicing variations. In a collection of RNA-seq data of 75 European individuals from the 1000 Genomes Project, we identified 506 ‘hidden’ personal splice junctions with polymorphic splice site dinucleotides that were supported by RNA-seq reads unmappable to the human reference genome. 437 of these splice junctions were novel, i.e. not in current human transcript annotations, and 94 were linked to genome-wide association study (GWAS) signals. Our data demonstrate that by mapping personal RNA-seq data to personal genomes, we can uncover numerous novel splicing variations in the human population, including those potentially underlying GWAS signals of complex human traits and diseases. We refer to our pipeline RNA-seq Personal Genome-alignment Analyzer (rPGA). The rPGA source code and user documents are freely available for download at https://github.com/Xinglab/rPGA.

## MATERIALS AND METHODS

### RNA-seq data sets

RNA-seq data for NA12891 were obtained from the NCBI Sequence Read Archive (SRR074943) (24). RNA-seq data for 75 European individuals were obtained from the Geuvadis RNA Sequencing Project (Supplementary Table S1; EBI ArrayExpress: E-GEUV-1) (23). All RNA-seq data were generated on lymphoblastoid cell lines (LCLs). Genotype data for these individuals were taken from the 1000 Genomes Browser (37). The genotype data were filtered to only include phased SNPs.

### RNA-seq read alignment

For each individual, the paired-end RNA-seq reads were aligned to hg19 and two haplotype versions (hap1 and hap2) of the personal genome using STAR (version 2.4.0f1) (35), generating three separate sets of alignment data. The maximum number of mismatches within reads was set to 0, the maximum number of multihits was set to 20 and the maximum intron size was set to 300 000. The minimum overhang length for splice junctions was set to 12 for GT/AG, GC/AG and AT/AC splice site dinucleotide motifs. Spliced alignments containing non-canonical splice junctions were not permitted. Truncated reads and reads with a truncated pair were considered unmapped.

### Identification of personal specific splice junctions

Hap1 specific junctions were splice junctions reported solely in the hap1 alignment data. Similarly, hap2 specific junctions were splice junctions reported solely in the hap2 alignment data and hap1hap2 specific junctions were splice junctions found in both the hap1 and hap2 alignment data but not in the hg19 alignment data. Personal specific splice junctions were defined to be the union of hap1, hap2 and hap1hap2 specific splice junctions. Likewise, splice junctions reported solely in the hg19 alignment data were called hg19 specific junctions, which can be used to assess the false positive rate of our procedure. Distinct reads were ones that had distinct starting alignment positions. We required personal specific splice junctions to be supported by ≥2 distinct reads for all further analyses.

### Splice site usage frequency

To compare the relative splicing activities at a personal specific splice site to nearby alternative splice sites, we calculated the splice site usage frequency using RNA-seq data. Note that frequencies were calculated only in the genome where the splice junction was identified. For example, a personal specific splice junction identified in one haplotype was only compared to all other junctions also found in that haplotype.

To calculate the relative usage frequency of personal splice junctions, we considered all Ensembl annotated splice junctions with overlapping genomic coordinates with our personal splice junction of interest. PSJ is the number of reads that span the personal specific splice junction and OSJ is the total number of reads that span all overlapping Ensembl annotated junctions. The relative usage frequency of the personal splice junction is PSJ/(PSJ + OSJ). We also performed this calculation for a subset of personal specific splice junctions that overlapped with only one other splice junction via alternative 5′ or 3′ splice site patterns. The number of reads supporting a splice junction was counted as the number of distinct splice junction spanning reads, i.e. reads with distinct genomic starting alignment positions.

### Association with GWAS signals

We obtained 207 889 GWAS SNPs with *P*-value < 10^−3^ from the GWASdb2 v4 catalog (38). We used PLINK v1.08p to calculate linkage disequilibrium (LD) correlations between splice site SNPs at personal specific splice junctions and all GWAS SNPs (39). The LD map was created using a CEU population and PLINK v1.07. We then identified splice site SNPs in high LD (*r*^2^ > 0.8) with GWAS SNPs.

### Linkage disequilibrium plot

Linkage disequilibrium (LD) plot for SNP *rs6948664* was generated using Haploview v4.2 (40). CEU LD data were obtained from the HapMap Project Genome Browser version E, using data release #28 (Phase II+III).

### Splice site score

Splice site (SS) scores (5′ or 3′ splice sites) were calculated using the MaxEntScan maximum entropy model (41). In cases where the personal SNP also affected the reference splice site, reference splice site sequences were changed accordingly to reflect the personal SNP. We defined Δ(SS score) = personal splice site score—reference splice site score, where Δ(SS score) > 0 corresponds to a personal splice site that is stronger than the alternative reference splice site.

### Experimental validation of personal specific splice junctions

HapMap LCLs were purchased from the Coriell Institute for Medical Research (Camden, NJ, USA). We prepared HapMap LCLs’ cDNAs as described before (42). To validate personal specific splice junctions, we designed a pair of polymerase chain reaction (PCR) primers targeting flanking constitutive exons of the novel splice junction. Regular PCR was carried out for 40 cycles. Final PCR products were purified by gel extraction and then confirmed by Sanger sequencing (Laragen, Inc, CA, USA). Primer sequences and HapMap individuals selected for PCR validation are shown in Supplementary Table S2.

## RESULTS

### A computational pipeline to identify personal splice junctions by mapping personal RNA-seq data to personal genomes

We developed a computational pipeline rPGA (Figure 1B) to discover hidden splice junctions by mapping personal RNA-seq data to the matching personal genome sequence. We applied this pipeline to analyze RNA-seq data from individuals with whole-genome genotype data in the 1000 Genomes project. Briefly, for each individual we modified the human reference genome (hg19) according to its genotype, resulting in two versions of personal genome sequences per individual (one for each haplotype), which we referred to as haplotype 1 (hap1) and haplotype 2 (hap2) in this manuscript. Next, an individual's RNA-seq reads were aligned to the human reference genome (hg19) and each of the two corresponding personal genomes (hap1 and hap2) using the RNA-seq alignment software STAR (35), resulting in three separate sets of alignment data per individual. We should note that STAR truncates a read once it reaches the maximum number of allowed mismatches or has too low of an alignment score (35). We considered such truncated reads as well as reads that did not align at all unmappable reads. We then remapped reads that were unmappable to hg19 to personal genomes (hap1 and hap2) to identify personal specific splice junctions (Figure 1). To constrain our analysis to the effect of splice site SNPs, we filtered out personal specific splice junctions with no SNP at the splice site dinucleotide motifs. 99.2% of filtered personal specific splice junctions were supported by reads that could have been aligned to the same location in the hg19 reference genome, but contained SNP(s) elsewhere along the reads that made them unmappable to hg19 due to the number of mismatches exceeding the aligner's threshold. To assess the rate of false positives from this pipeline, we reversed the alignment procedure by re-aligning reads unmappable to personal genomes (hap1 and hap2) to hg19 to identify hg19-specific splice junctions. We consider such hg19-specific splice junctions false positives, because their supporting splice junction reads were generated from the personal genome sequence of the individual.

As a proof-of-concept analysis using this pipeline, we initially performed personal genome RNA-seq alignment using RNA-seq data of a male Caucasian (NA12891) (24). We identified 33 personal specific splice junctions, including 24 novel splice junctions not documented in current human transcript annotations (Ensembl version #75). The reverse mapping procedure (see above) identified only 3 hg19-specific splice junctions, suggesting that our pipeline has low false positives. We also checked whether our pipeline could identify known alternative splicing variations arising from personal specific splice junctions. Indeed, we successfully captured a known personal specific 3′ splice site of intron 20 in *NPHP4* associated with SNP *rs1287637* (*T > A* on the RNA sense strand which created an ‘AG’ 3′ splice site). *NPHP4* is involved in renal function and its mutations are known to cause juvenile end stage renal disease (43). The personal splice junction is supported by 5 distinct reads, though it is unlikely to be identified through traditional reference genome based RNA-seq mapping because the supporting reads are unmappable to hg19 due to the lack of canonical splice site dinucleotides in the reference genome. Interestingly, the T allele in the reference genome causes activation of two downstream 3′ splice sites and is associated with alternative transcript isoforms with 6 nt and 42 nt deletions of exon 21. Individuals carrying the ‘*T*’ allele of SNP *rs1287637* (i.e. *A/T* or *T/T* genotype) have reduced renal function manifested as decreased glomerular filtration rate (43). This example shows that our strategy can identify personal specific splice junctions that would otherwise be missed when mapping RNA-seq reads to the reference human genome.

### Comprehensive identification of personal specific splice junctions in 75 European individuals

**Figure 2.**
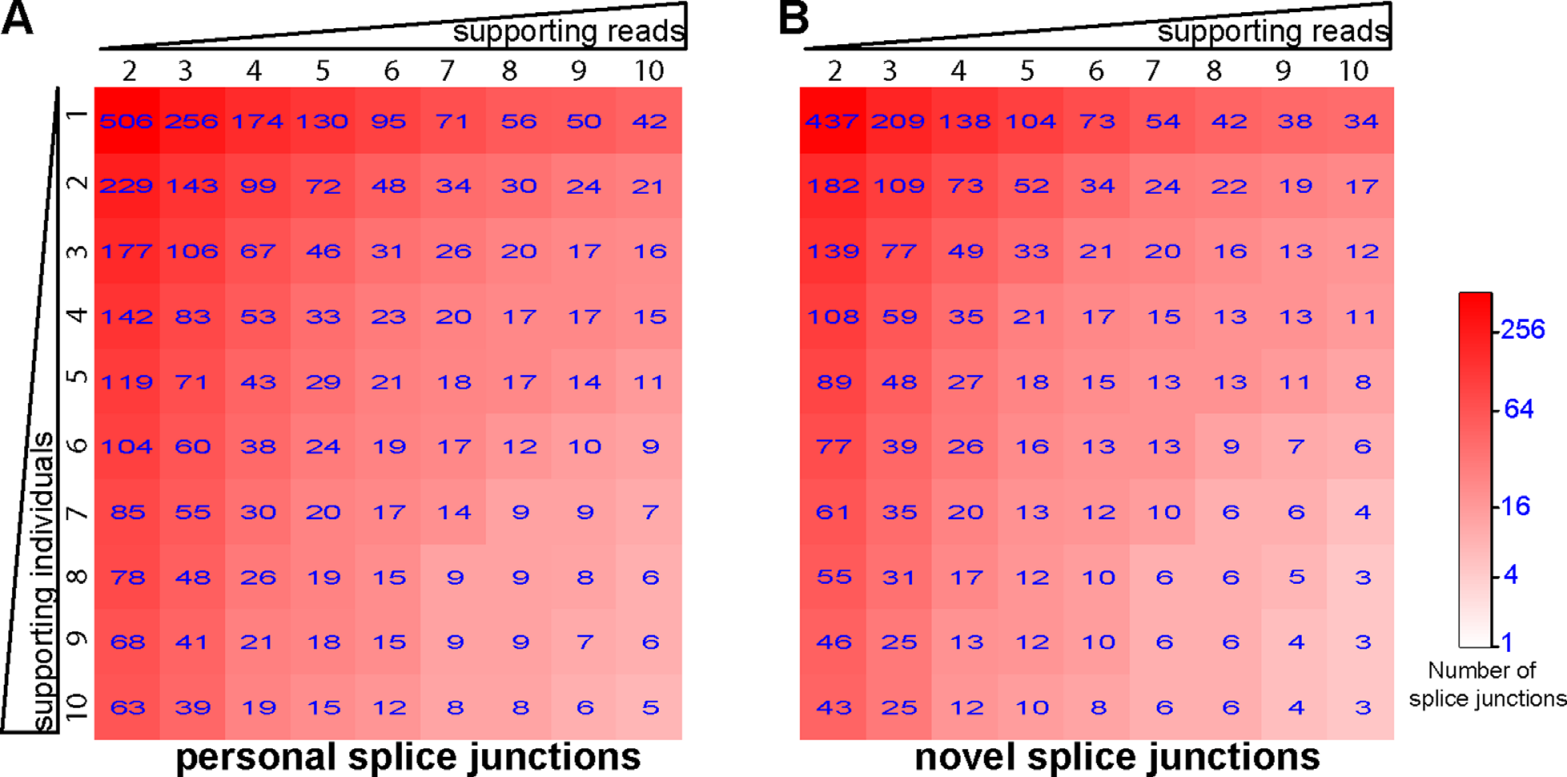
Number of personal specific splice junctions supported by different numbers of RNA-seq reads and individuals. Heatmap of **(A)** the total number of personal specific splice junctions, and **(B)** the total number of novel personal specific splice junction identified across 75 CEU individuals. Columns represent an increasing requirement for the minimum number of supporting splice junction reads. Rows represent an increasing requirement for the minimum number of supporting individuals.

Next, we expanded our analysis to RNA-seq data of 75 European individuals. These data were obtained from the Geuvadis RNA sequencing project on 1000 Genomes samples (23) (see Materials and Methods). We identified a total of 506 distinct personal specific splice junctions among the 75 individuals (Figure 2A and Supplementary Table S3), with at least two distinct supporting RNA-seq reads in at least one individual. The reverse alignment procedure identified only 27 hg19-specific splice junctions. In fact, at the same threshold of supporting evidence, the number of hg19-specific splice junctions was always at most a few percent of the number of personal specific splice junctions (see Supplementary Figure S1), suggesting that the false positive rate of our pipeline was consistently low even for splice junctions identified in only a single individual. We also compared our results to the Ensembl transcript annotations (version #75) (44) to classify personal specific splice junctions as either known or novel. The 506 splice junctions included 69 known and 437 novel splice junctions (Figure 2B). Our list of 69 known personal specific splice junctions includes events with documented disease relevance. For example, a known personal specific splice junction in *IRF5* was identified in 25 individuals. Splice site SNP *rs2004640* (*G > T* on the RNA sense strand) creates an alternative ‘GT’ 5′ splice site to enable the splicing of exon 1B as an alternative first exon (45). Interestingly, *IRF5* has four alternative first exons, all of which are in the 5′-UTR (46). Exon 1B is the only alternative first exon with a p53-binding site in the associated promoter region (47). Furthermore, the T allele of *rs2004640* has 2.7-fold higher *IRF5* mRNA level (46). Overexpression of *IRF5* is associated with susceptibility of autoimmune diseases, including systemic lupus erythematous, rheumatoid arthritis and multiple sclerosis (48,49). A second example is a known splice junction in *CPNE1* identified in 9 individuals. An exonic SNP *rs2425068* (*A > G*) immediately downstream of the canonical 3′ splice site creates a pair of tandem NAGNAG 3′ splice sites (*CAGCAA > CAGCAG*) (50). Use of the downstream 3′ splice site results in one amino acid 413Q deletion in the VWFA (Von Willebrand Factor Type A) domain. This SNP is associated with plasma protein C levels and potentially venous thromboembolism (51).

We next asked whether these ‘hidden’ personal splice junctions could be expressed at a high enough level to be considered more than just baseline splicing noise. For this analysis, we calculated the relative usage frequency of the identified personal splice junctions using RNA-seq data (see Materials and Methods). As expected, personal splice junctions had a wide range of usage frequencies. 460, 311 and 193 of the 506 personal splice junctions had an average usage frequency no less than 5%, 15% and 50%, respectively, when detected. For example, SNP *rs2004640* (*G > T*) in *IRF5* as mentioned above had average personal splice junction usage frequency of ≈11% in 25 individuals, while SNP *rs2425068* (*A > G*) in *CPNE1* had average personal splice junction usage frequency of ≈65% in 9 individuals. We also calculated the detection frequency of personal splice junctions across individuals, as the percentage of individuals with a given personal splice junction detected among all individuals carrying the splice site SNP. We found that 323, 218 and 108 personal splice junctions had a detection frequency no less than 5%, 15% and 50%, respectively, among individuals carrying the splice site SNPs. These results indicate that personal specific splice junctions arising from splice site polymorphisms can be used in a significant portion of the final transcript products within the population.

We performed an in-depth analysis to assess how the usage frequency of personal specific splice junctions was correlated with the strength of their associated splice sites. To remove potential confounding factors and obtain meaningful estimates of splice junction usage frequencies, we imposed several additional requirements: first, the personal specific splice junction must form alternative 5′ or 3′ splice sites with exactly one other reference splice junction expressed in the personal transcriptome; second, the personal and reference splice junctions must have a minimum combined read count of 10 distinct RNA-seq reads to ensure a high enough coverage for estimating splice junction usage frequencies; and third, individuals must be homozygous for the splice site SNP because relative splice junction frequency in the RNA-seq data is dependent on the genotype of the individual. We identified a total of 149 instances of personal specific splice junctions that met these criteria. Among them, 77 had weaker splice sites compared to the reference splice sites, and 72 had stronger splice sites compared to the reference splice sites. We found that personal splice junctions with stronger splice sites had significantly higher usage frequencies than those with weaker splice sites (mean frequency 36.2% versus 19.7%; *P* = 5.5 × 10^−4^, Wilcoxon test) (Supplementary Figure S2).

### Experimental validation of personal specific splice junctions

We used PCR followed by Sanger sequencing to perform experimental validation of novel personal specific splice junctions. PCR analyses were carried out in lymphoblastoid cell lines (LCLs) carrying the personal specific splice junctions of interest. To facilitate PCR primer design and analysis, we randomly selected eight novel personal specific splice junctions which had alternative 5′ or 3′ splice site patterns with at least one other annotated splice site and a usage frequency of at least 10% in the corresponding individuals. A pair of PCR primers targeting flanking constitutive exons was designed for each novel personal specific splice junction. Putative PCR amplicons were further confirmed by Sanger sequencing. All eight novel splice junctions were validated (Table 1). We validated novel personal specific splice junctions present in as few as 1 individual and as many as 69 individuals.

**Table 1.** Experimental validation of novel personal specific splice junctions

Gene symbol	Genomic coordinates (hg19)	Average relative usage frequency	Frequency standard deviation	# Individuals supported	GWAS disease/trait	Validated	novel
*ANXA6*	chr5:150483256–150484805	0.48	0	1		Yes	Yes
*ARSG*	chr17:66352945–66364691	0.82	0.18	15		Yes	Yes
*ASMTL*	chrX:1540735–1544272	0.10	0	1		Yes	Yes
*DHRS12*	chr13:52345636–52345956	0.23	0	1	Coronary Artery Disease (72)	Yes	Yes
*GRAMD1A*	chr19:35505291–35506730	0.65	0.08	43		Yes	Yes
*NPNT*	chr4:106816880–106819054	0.65	0.28	9		Yes	Yes
*OAS1*	chr12:113355505–113357194	0.21	0.07	69	Multiple complex diseases (73)	Yes	Yes
*U2AF1L4*	chr19:36233704–36234652	0.28	0.09	6		Yes	Yes

**Figure 3.**
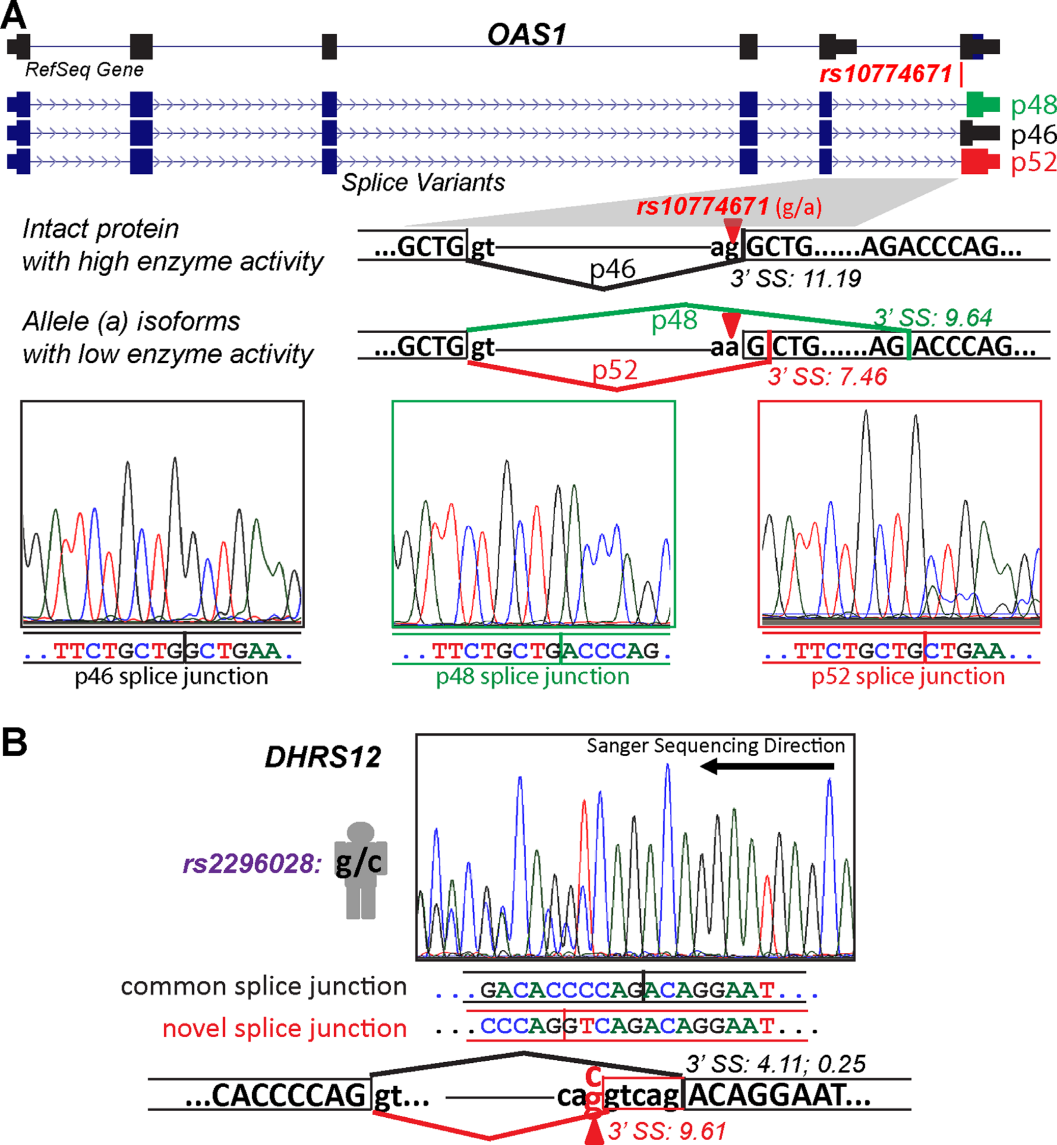
Experimental validation and sequencing chromatograms of personal specific splice junctions in *OAS1* and *DHRS12*. **(A)** SNP *rs10774671* creates a personal 3′ splice site of *OAS1*. The reference 3′ splice site produces an intact protein isoform p46. The SNP *rs10774671* (*G* to *A*) abolishes the reference 3′ splice site, resulting in the usage of a personal 3′ splice site and an internal cryptic 3′ splice site corresponding to alternative protein isoforms p52 and p48 with reduced enzyme activity. **(B)** SNP *rs2296028* creates a personal 3′ splice site 5 nt upstream of the reference 3′ splice site of *DHRS12* exon 8. This SNP also decreases the score of the reference 3′ splice site from 4.11 to 0.25. 3′ SS: 3′ splice site.

One example of validated splice junctions is a personal splice junction of exon 6 of *OAS1*, observed in RNA-seq data of 69 individuals. *OAS1* encodes 2′-5′-oligoadenylate synthetase and is involved in viral and endogenous RNA degradation to inhibit viral replication (52). Splice site SNP *rs10774671* (*G > A*) creates a cryptic 3′ splice site that is shifted 1 nt downstream from the canonical 3′ splice site in the reference genome. Simultaneously, *rs10774671* corrupts the reference 3′ splice site (*AG > AA*) as shown in Figure 3A. Usage of the personal splice junction is predicted to produce a protein isoform (i.e. p52) with reduced enzyme activity and can thus affect immune response to viral infection (53).

A second example is a novel personal specific splice junction in exon 8 of *DHRS12* that was identified in one individual (Figure 3B). *DHRS12* codes for an enzyme in the short-chain dehydrogenases/reductases (SDR) family. Although *DHRS12* itself is not a well-characterized gene, it is known that SDRs in general are responsible for metabolizing substances in the body including hormones and xenobiotics (54–56). Mutations in these genes have been associated with a variety of metabolic disorders (57). Splice site SNP *rs2296028* (*C > G* on the RNA sense strand) creates a stronger, novel 3′ splice site (splice site score = 9.61 by MAXENT (41)) 5 nt upstream from the reference 3′ splice site (score = 4.11) (Figure 3B). Moreover, the G allele of SNP *rs2296028* decreases the reference 3′ splice site score from 4.11 to 0.25. RNA-seq data as well as our PCR and sequencing data indicate that this much stronger novel 3′ splice site is used in one individual heterozygous for the splice site SNP.

We should note that these eight validated splice junctions were randomly selected from our identified novel personal specific splice junctions, rather than being cherrypicked from the most frequently expressed and identified novel splice junctions. Our independent experimental validation confirmed the existence of hidden personal splicing variations that would otherwise be overlooked by mapping personal RNA-seq reads to the reference genome.

### Personal specific splice junctions are linked to GWAS signals of complex traits and diseases

Finally, we investigated whether our discovered personal specific splice junctions were associated with GWAS signals of complex traits and diseases. GWAS studies have identified an abundance of associations between genomic variants and phenotypes. Interpreting the growing catalog of GWAS signals is a powerful application for elucidating human transcriptome variation (23). However, more often than not, GWAS signals merely serve as markers for phenotype association and reveal little regarding the underlying causal genomic variants as well as the molecular mechanisms for phenotype variability or disease pathogenesis (58). Although initial analyses of GWAS signals focused on those in protein coding regions, recent evidence suggests that many functional variants lie in non-coding regions and affect phenotypes through regulatory mechanisms (59). As alternative splicing plays a powerful role in transcriptome variation and phenotype diversity (13), personal specific splice junctions arising from splice site SNPs are likely candidates for functional causal variants associated with GWAS signals. To investigate this, we sought to identify all personal splice junction associated splice site SNPs in high (*r*^2^ > 0.8) LD with GWAS signals listed in GWASdb2 v4 (see Materials and Methods) (38). GWASdb integrates a number of well-established collections of GWAS SNPs, including the commonly used NHGRI GWAS catalog (60). We identified 9 known and 85 novel personal specific splice junctions with splice site SNPs linked to GWAS signals (Supplementary Table S4 and Table 2). The list includes our experimentally validated splice junctions in *OAS1* and *DHRS12* (described above), as well as other personal splice junction associated SNPs with potential medical significance.

**Table 2.** Selected list of personal specific splice junction SNPs linked to GWAS signals

Gene symbol	Genomic coordinates (hg19)	Novel	Splice site SNP	Linked GWAS SNP(s)	GWAS gene symbol	GWAS disease/trait	Reference
*AHRR*	chr5:428122–430060	Yes	*rs72717415*	*rs12188164*	*AHRR*	Cystic fibrosis severity	(74)
*ALG8*	chr11:77838483–77850518	Yes	*rs10793289*	*rs10899440*	*ALG8*	Endometriosis	(75)
*ATP5A1*	chr18:43671818–43673144	Yes	*rs8083998*	*rs13381709, rs7244921, rs8089150*	*ATP5A1*	HIV-1 disease progression	(76)
*DHRS12*	chr13:52345636–52345956	Yes	*rs2296028*	*rs2296028*	*DHRS12*	Coronary artery disease	(72)
*ICA1*	chr7:8167773–8168380	Yes	*rs6948664*	*rs4725072*	*ICA1*	Systemic lupus erythematosus and Systemic sclerosis	(67)
*NBR2*	chr17:41290940–41291953	Yes	*rs11657835*	*rs11655505*	*BRCA1*	Breast Neoplasms	(77)
*NDUFAF6*	chr8:95988208–95993082	Yes	*rs6983948*	*rs16893776*		Blood pressure	(78)
*OAS1*	chr12:113355506–113357194	Yes	*rs10774671*	*rs2660*	*OAS1*	Multiple complex diseases	(73)
*PPP1R3B*	chr8:8999187–9008072	Yes	*rs330924*	*rs330911*	*PPP11R3B*	Alzheimer's disease (late onset)	(79)
*STYXL1*	chr7:75630274–75633075	Yes	*rs8565*	*rs6978677*	*STYXL1*	Lymphocyte counts	(80)

**Figure 4.**
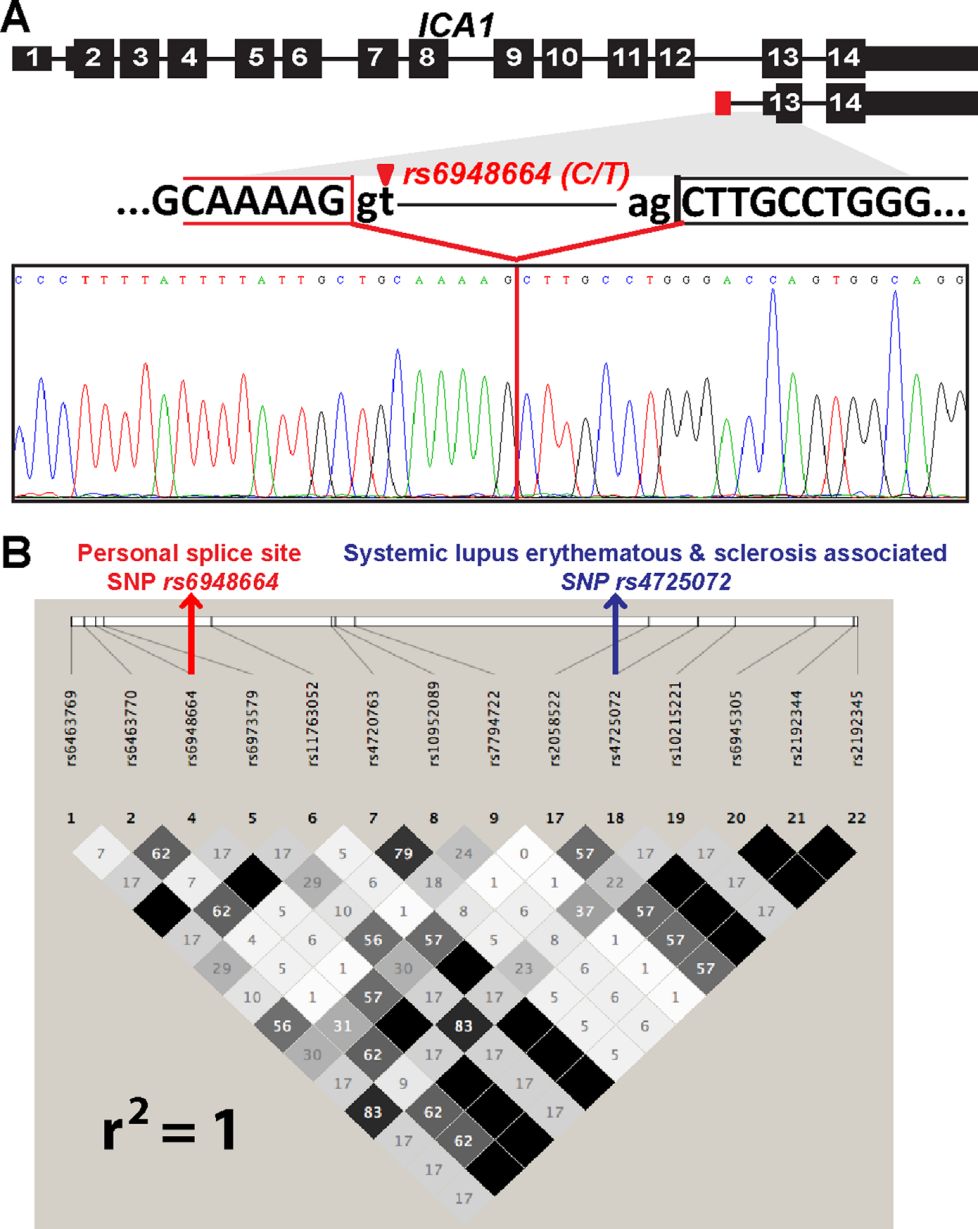
A personal specific splice junction of *ICA1* is linked to GWAS signals of diseases. **(A)** The schematic gene structure and sequencing chromatogram of the novel personal specific splice junction in *ICA1*. SNP *rs6948664* creates a novel personal 5′ splice site in intron 12 of *ICA1*, resulting in a novel alternative first exon of *ICA1*. **(B)** Linkage disequilibrium (LD) plot of the CEU population indicates that the personal specific splice junction SNP *rs6948664* is in perfect LD with the GWAS SNP of systemic lupus erythematous and sclerosis *rs4725072* (*r*^2^ = 1).

A highly intriguing example is a personal specific splice junction in *ICA1*. *ICA1* encodes autoantigen ICA69, which is involved in vehicular transport of insulin secretory granule proteins and is known to play an important role in autoimmune diseases, including type 1 diabetes, rheumatoid arthritis and Sjogren's syndrome (61–64). SNP *rs6948664* (*C > T*) activates a novel 5′ splice site (*GC > GT*, 5′ splice site score = 8.4) in intron 12 of *ICA1*. Our RNA-seq alignment indicates that this novel 5′ splice site is paired with the downstream 3′ splice site of exon 13. However, in the RNA-seq data we did not find any splice junction reads from exon 12 or other upstream exons into the potential novel exon within intron 12, raising the possibility that this novel personal specific splice junction may be associated with a novel alternative first exon within intron 12. To test this hypothesis, we designed a pair of PCR primers targeting exon 12 and exon 13 of *ICA1*, and a separate pair of PCR primers targeting the potential novel exon in intron 12 and exon 13. As expected, the novel personal specific splice junction was only observed in individuals carrying the *T* allele of *rs6948664* (Figure 4). Moreover, the primer pair targeting the two flanking exons (exon 12 and 13) failed to amplify any PCR amplicon that contained the potential novel exon in intron 12, suggesting that the novel personal specific splice junction is not paired with an upstream splice junction that creates a novel internal exon of *ICA1*. Thus, we conclude that *rs6948664* activates a novel alternative first exon in *ICA1*. Of note, the use of this novel alternative first exon is expected to produce a much shorter mRNA isoform (≈1100nt), compared to the full-length mRNA transcript (2473 nt, NM_001136020), and the longest putative open reading frame of the novel transcript is only 75aa. The predicted 75aa protein isoform has the same reading phase as the canonical mRNA isoform but loses the AH domain, which has been shown to dimerize and bind to Arf and Rho family GTPases (65,66). Arf and Rho GTPases are required for regulation of many cellular processes including cell motility and Golgi function (65). The novel personal specific splice junction was identified in 23 individuals, with an average usage frequency of 59%. Additionally, *rs6948664* is in complete LD (*r*^2^ = 1) with GWAS signal *rs4725072*, which has been identified as significantly associated with systemic lupus erythematous (SLE) and systemic sclerosis (38,67). However, no underlying causal variants were identified for SLE or systemic sclerosis in the original GWAS study. Interestingly, individuals showing RNA-seq evidence for the novel personal specific splice junction have significantly higher *ICA1* gene expression levels in LCL cells compared to those without (*P* = 7 × 10^−3^, Wilcoxon test). It has been found that overexpression of *ICA1* in insulinoma INS-1 cells impairs secretory granule protein transport (61). Collectively, our data suggest that *rs6948664* is a likely causal variant for SLE or systemic sclerosis by significantly affecting *ICA1*'s expression level and/or protein output. We note that recent work has described the roles of first exon and 5′ splice site in transcriptional regulation (68–70), which could underlie our observed association between this alternative first exon and *ICA1* gene expression level. However, we cannot rule out the possibility that this observation could be due to the simple fact that it is more likely to detect such an alternative mRNA isoform by RNA-seq when the overall gene expression level is high. Further studies are needed to assess the causal impact of this novel alternative first exon on *ICA1* gene transcription and function.

Of all GWAS-associated personal splice junctions, 63 formed alternative 5′ or 3′ splice sites with reference splice junctions. 40 of these 63 GWAS-associated personal splice junctions (63.5%) were potentially frame-shifting because the distance between the personal and reference splice sites was not an exact multiple of three nucleotides. The percentage was similar in non-GWAS-associated personal splice junctions.

## DISCUSSION

Dissecting the molecular mechanisms of transcriptome variation in human populations is critical for understanding the biology of complex traits and diseases. Alternative splicing is a major contributor to transcriptome variation and phenotypic diversity among human individuals (9,10,13). Thus, identifying splicing variations in human populations will shed light on the genetic basis of diseases. A number of recent studies have used RNA-seq to characterize genetic variation of alternative splicing in human cell lines and tissues (22–28). These studies typically generate quantitative estimates of alternative splicing using RNA-seq reads aligned to a single reference genome. However, as we demonstrated in this work, important splicing variations may be missed by mapping personal RNA-seq reads to the reference genome.

In this study we implemented a computational pipeline rPGA (RNA-seq Personal Genome-alignment Analyzer; https://github.com/Xinglab/rPGA) to identify splicing variations in individual transcriptomes, by mapping personal RNA-seq reads to personal genomes. The importance of using personal genome information has been discussed previously for RNA-seq studies, mostly for the purpose of reducing allelic bias in mapping personal RNA-seq reads to the reference genome (30–32). Here we investigated a distinct issue in RNA-seq alignment, namely the identification of novel, personal specific splice junctions from personal RNA-seq data. Because commonly used RNA-seq aligners all rely on the consensus splice site dinucleotide motifs to map reads across splice junctions, if a genetic polymorphism creates a novel splice site dinucleotide motif, the resulting splice junction reads utilizing this novel splice site will likely be unmappable to the reference genome by a standard RNA-seq aligner. Indeed, using a personal genome alignment approach, we identified many novel personal specific splice junctions at a low false positive rate (Figure 2). Moreover, eight novel splice junctions selected for experimental testing were all validated by RT-PCR and sequencing. Thus, our results indicate that we could uncover ‘hidden’ splicing variations in individual transcriptomes by aligning personal RNA-seq reads to personal genomes. We should clarify that not all personal specific splice junctions are due to SNPs at the splice site dinucleotide motifs, because other exonic or intronic SNPs can also affect splicing. However, such differential splicing events could readily be detected by conventional RNA-seq alignment procedures and sQTL detection algorithms (26,71) and are not the focus of this work.

We identified 506 personal specific splice junctions in an RNA-seq data set of 75 European individuals, among which 437 were novel splice junctions not documented in current human transcript annotations (Ensembl version #75). 94 splice junctions had splice site SNPs associated with GWAS signals of human traits and diseases. These involve genes whose splicing variations have been implicated in diseases (such as *OAS1*, Figure 3A), as well as novel associations between alternative splicing and disease (such as *ICA1*, Figure 4). To put these numbers in a proper context, as a comparison our previous analysis of alternative splicing in 41 European individuals identified 140 splicing QTLs from the RNA-seq data, including 10 linked to GWAS SNPs (26). Thus, this personal genome approach to RNA-seq read alignment allows us to tap into a large but previously unknown catalog of splicing variations in human populations, and should be recommended as a routine step for secondary analyses of RNA-seq data with matching genome information. Given that parallel sequencing of DNA and RNA has become a popular strategy for genomic studies of diseases, this approach may also be useful for discovering splicing variations in diseased tissues with RNA-seq data matched with exome sequencing or genome sequencing data. For example, this approach may help identify novel cancer-specific splicing variations arising from somatic mutations that create splice site dinucleotide motifs in cancer genomes. It is also possible to extend the rPGA pipeline to identify personal specific splice junctions arising from other types of genomic variants, such as indels or polymorphic structural variations.

## Supplementary Material

SUPPLEMENTARY DATA
