# Supplementary material for: Discover hidden splicing variations by mapping personal transcriptomes to personal genomes
Source: Nucleic Acids Res. 2015 Nov 17;43(22):10612–22. doi: 10.1093/nar/gkv1099 (PMC4678817; doi:10.1093/nar/gkv1099)
Supplement: SUPPLEMENTARY DATA [file supp_43_22_10612__index.html]

Discover hidden splicing variations by mapping personal transcriptomes to personal genomes — SUPPLEMENTARY DATA 

# Discover hidden splicing variations by mapping personal transcriptomes to personal genomes

## SUPPLEMENTARY DATA

- SUPPLEMENTARY DATA
- SUPPLEMENTARY DATA
- SUPPLEMENTARY DATA
- SUPPLEMENTARY DATA
- SUPPLEMENTARY DATA
