# Supplementary material for: Discover hidden splicing variations by mapping personal transcriptomes to personal genomes
Source: Nucleic Acids Res. 2015 Nov 17;43(22):10612–22. doi: 10.1093/nar/gkv1099 (PMC4678817; doi:10.1093/nar/gkv1099)
Supplement: SUPPLEMENTARY DATA [file supp_gkv1099_nar-02506-n-2015-File006.docx]

**Figure S1:** **Number of hg19-specific splice junctions.**

The total number of hg19-specific splice junctions, which were identified in the hg19 alignment but not in the personal alignment. Columns represent an increasing requirement for the minimum number of supporting splice junction reads. Rows represent an increasing requirement for the minimum number of supporting individuals.


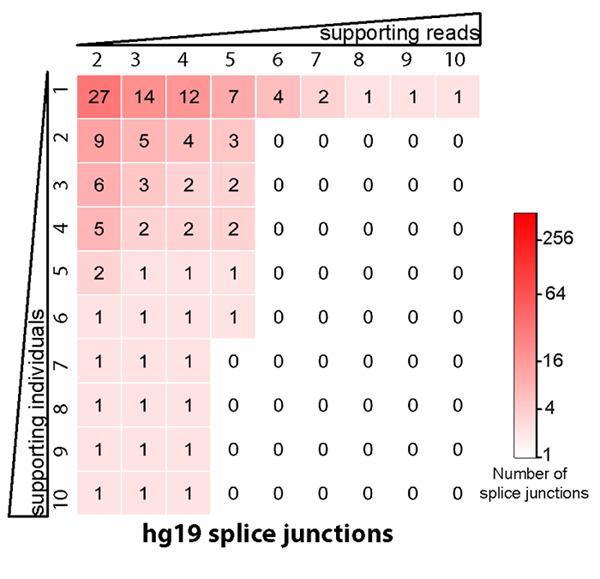


**Figure S2:** **Average usage frequency versus splice site score of personal specific splice junctions.**

Relative usage frequency of strong (∆(SS score) > 0) and weak (∆(SS score) ≤ 0) personal specific splice junctions. The middle line represents the median; the top and bottom lines of the rectangle represent the 3^rd^ and 1^st^ quartiles, respectively; the top whisker represents the 3^rd^ quartile plus 1.5 times the interquartile range; the bottom whisker represents the 1^st^ quartile minus 1.5 times the interquartile range; The notches represent the 95% confidence interval around the median. SS, splice site. ∆(SS score) = personal splice site score – reference splice site score.

**
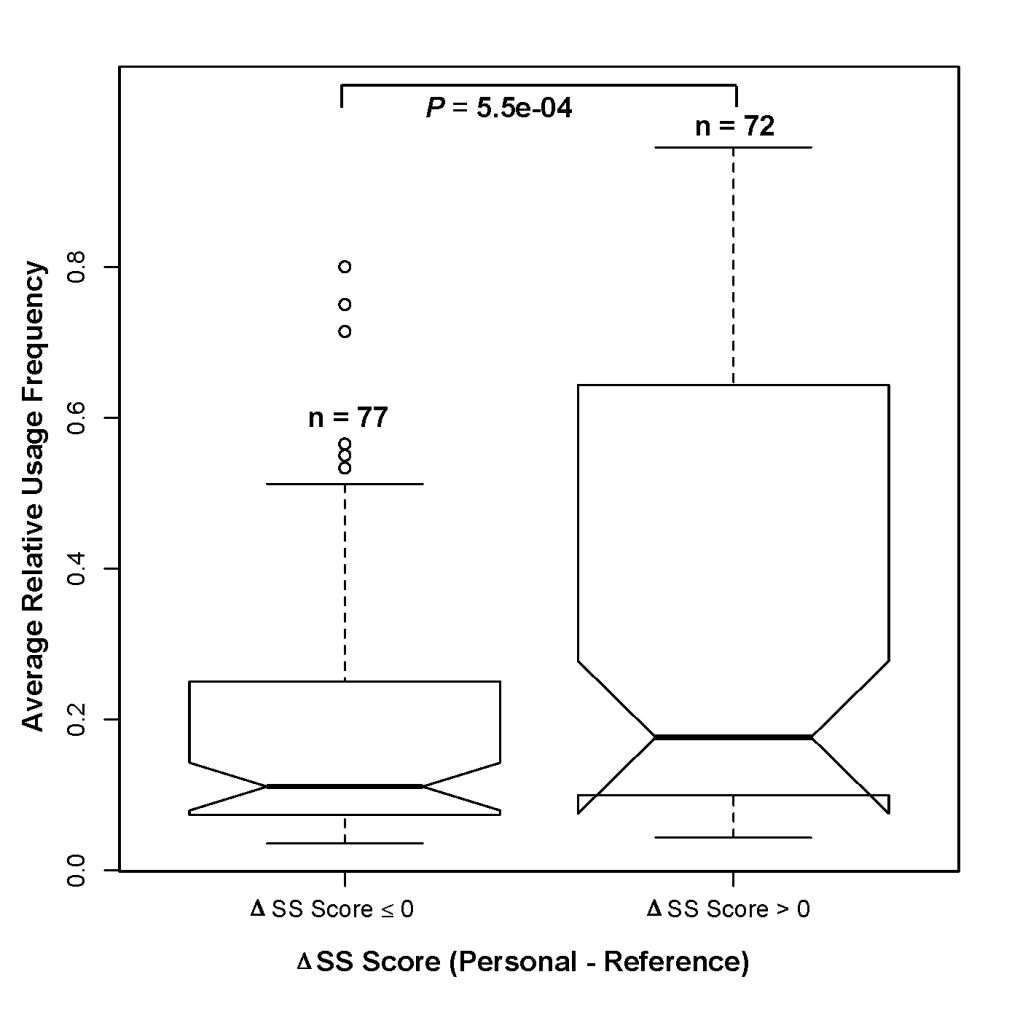
**
